# Prediction algorithm for ICU mortality and length of stay using machine learning

**DOI:** 10.1038/s41598-022-17091-5

**Published:** 2022-07-28

**Authors:** Shinya Iwase, Taka-aki Nakada, Tadanaga Shimada, Takehiko Oami, Takashi Shimazui, Nozomi Takahashi, Jun Yamabe, Yasuo Yamao, Eiryo Kawakami

**Affiliations:** 1 Department of Emergency and Critical Care Medicine, Chiba University Graduate School of Medicine, 1-8-1 Inohana, Chuo-ku, Chiba, Chiba 260-8677 Japan; 2 Smart 119 Inc., 7th floor, Chiba Chuo Twin Building No. 2, 2-5-1 Chuo, Chiba, Japan; 3 Department of Artificial Intelligence Medicine, Chiba University Graduate School of Medicine, Chiba, Japan; 4 Medical Sciences Innovation Hub Program, RIKEN, 1-7-22 Suehiro-cho, Tsurumi-ku, Yokohama, Kanagawa Japan

**Keywords:** Medical research, Predictive markers, Prognostic markers

## Abstract

Machine learning can predict outcomes and determine variables contributing to precise prediction, and can thus classify patients with different risk factors of outcomes. This study aimed to investigate the predictive accuracy for mortality and length of stay in intensive care unit (ICU) patients using machine learning, and to identify the variables contributing to the precise prediction or classification of patients. Patients (n = 12,747) admitted to the ICU at Chiba University Hospital were randomly assigned to the training and test cohorts. After learning using the variables on admission in the training cohort, the area under the curve (AUC) was analyzed in the test cohort to evaluate the predictive accuracy of the supervised machine learning classifiers, including random forest (RF) for outcomes (primary outcome, mortality; secondary outcome, length of ICU stay). The rank of the variables that contributed to the machine learning prediction was confirmed, and cluster analysis of the patients with risk factors of mortality was performed to identify the important variables associated with patient outcomes. Machine learning using RF revealed a high predictive value for mortality, with an AUC of 0.945 (95% confidence interval [CI] 0.922–0.977). In addition, RF showed high predictive value for short and long ICU stays, with AUCs of 0.881 (95% CI 0.876–0.908) and 0.889 (95% CI 0.849–0.936), respectively. Lactate dehydrogenase (LDH) was identified as a variable contributing to the precise prediction in machine learning for both mortality and length of ICU stay. LDH was also identified as a contributing variable to classify patients into sub-populations based on different risk factors of mortality. The machine learning algorithm could predict mortality and length of stay in ICU patients with high accuracy. LDH was identified as a contributing variable in mortality and length of ICU stay prediction and could be used to classify patients based on mortality risk.

## Introduction

Critically ill patients have a potential risk of death. To prevent this, substantial data including physiological parameters and laboratory tests are collected on intensive care unit (ICU) admission and throughout the ICU stay. Based on the integral evaluation using these data, health professionals, including physicians can provide care and predict clinical outcomes. Death is the primary clinical outcome, while length of ICU stay, which potentially reflects severity of the survivors and alters medical costs, may be another key outcome. Precise prediction of clinical outcomes at an early time point may contribute to improving the quality of patient care, medical resources/costs, and clinical outcomes^1,2^.

Recent advances in machine learning have led to an increase in the development of prediction algorithms using machine learning approaches in critical care^3^. A recent systematic review reported that a machine learning approach could be used to develop precise prediction algorithms for mortality in ICU patients compared to conventional statistical approaches (patients n > 10,000, area under the curve [AUC] 0.89-0.94)^4,5^. While high mortality precision has been reported, analysis results to identify important variables in mortality prediction algorithms have not been sufficiently reported. Other clinical outcomes besides mortality, including length of ICU stay, are rarely analyzed. In addition to prediction, clustering into sub-populations is another strength of the machine learning approach; however, studies on clustering critically ill patients are limited.

Thus, we hypothesized that machine learning approaches could be used to develop precise prediction algorithms for ICU mortality and length of ICU stay, and that machine learning analysis could identify important variables for outcomes, while clustering may clarify subcategories. We collected a large data set on ICU admission from a single-center surgical/medical mixed ICU and analyzed three types of machine learning approaches.

## Results

### Prediction of ICU mortality

The entire cohort of 12,747 patients was randomly split into a training cohort of 10,197 patients (80%) and a test cohort of 2,550 patients (20%) (Table 1, Supplementary Table S1). The number of ICU survivors was 12,133 (95.2%). Patient background and outcome were comparable between the training and test cohort data, except for the fraction of elective operations (P = 0.04).

**Table 1:** Baseline characteristics and outcomes of patients in the training and test cohorts.

Variables	Training cohort	Test cohort	P-value
	(n = 10,197)	(n = 2550)	
**Demographic data**			
Age, years	67 (53–75)	67 (54–75)	0.41
Missing	5 (0.0)	1 (0.0)	0.84
Male	6366 (62.4)	1567 (61.5)	0.36
Missing	1 (0.0)	1 (0.0)	0.29
**Diagnosis on admission**			
Sepsis/Septic shock	411 (4.0)	98 (3.8)	0.67
Post cardiac arrest syndrome	284 (2.8)	67 (2.6)	0.66
Stroke	151 (1.5)	41 (1.6)	0.64
Acute coronary syndrome	305 (3.0)	83 (3.3)	0.49
Heart failure	426 (4.2)	89 (3.5)	0.12
Trauma	222 (2.2)	61 (2.4)	0.51
Others	4579 (44.9)	1130 (44.3)	0.59
Missing	3819 (37.5)	981 (38.5)	0.19
**Admission route**			
Emergency room	1123 (11.0)	287 (11.3)	0.73
General ward	689 (6.8)	170 (6.7)	0.87
Operating room, emergency	241 (2.4)	59 (2.3)	0.89
Operating room, elective	2765 (27.1)	639 (25.1)	0.04
Other hospital	30 (0.3)	13 (0.5)	0.15
Missing	5349 (52.5)	1382 (54.2)	0.12
**Comorbidity**			
Acquired immunodeficiency syndrome	6 (0.1)	0 (0.0)	0.22
Hematological diseases*	29 (0.3)	7 (0.3)	0.93
Heart failure	361 (3.5)	80 (3.1)	0.32
Lymphoma	22 (0.2)	7 (0.3)	0.58
Respiratory failure	443 (4.3)	94 (3.7)	0.14
Metastasis	99 (1.0)	22 (0.9)	0.62
Immunosuppression	170 (1.7)	41 (1.6)	0.83
Liver failure	54 (0.5)	17 (0.7)	0.41
Cirrhosis	33 (0.3)	8 (0.3)	0.94
Dialysis	113 (1.1)	29 (1.4)	0.90
Missing^†^	0 (0.0)	0 (0.0)	
APACHE II score	20 (14–28)	19 (14–27)	0.16
SOFA score	5 (2–8)	4 (2–7)	0.13
**Outcomes**			
Survival discharge	9703 (95.2)	2430 (95.3)	0.77
**Length of ICU stay**			
≤ 1 week	8758 (90.3)	2216 (91.2)	0.16
1–2 weeks	549 (5.7)	121 (5.0)	0.19
> 2 weeks	396 (4.1)	93 (3.8)	0.57

Data are expressed as median (interquartile range) for continuous variables and as exact numbers (%) for categorical variables.

*APACHE* acute physiology and chronic health evaluation, *SOFA* sequential organ failure assessment, *ICU* intensive care unit.

P-values were calculated using Pearson’s chi-square test and Mann–Whitney U test.

*Hematological diseases include acute myeloid leukemia and multiple myeloma.

^†^Comorbidities were treated as not having if their data were missing.

**Figure 1:**
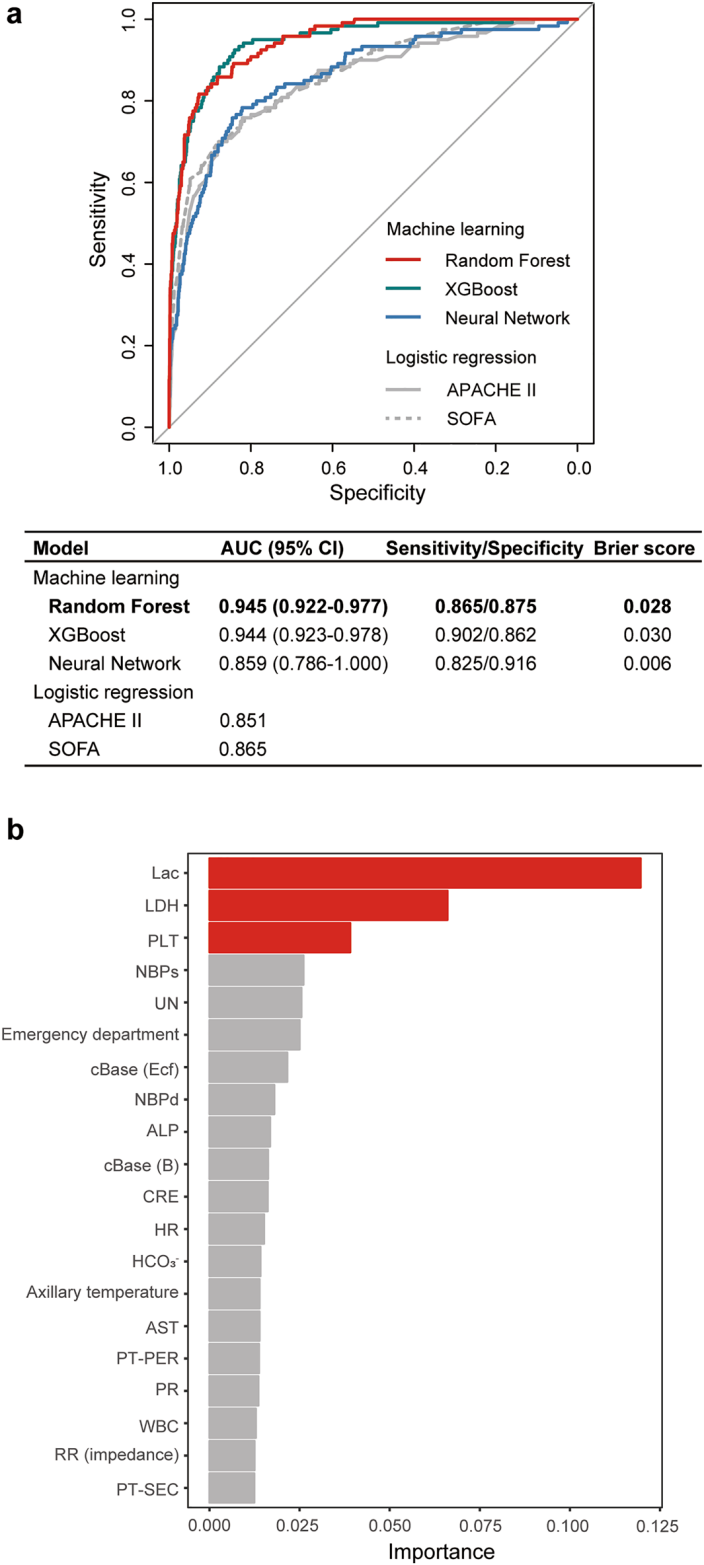
Predictive accuracy and key variables for intensive care unit mortality in the test cohort. (**a**) ROC curves and AUCs for ICU mortality were obtained from machine learning methods (Random Forest, XGBoost and Neural Network) and logistic regression. (**b**) Relative importance of variables for ICU mortality in Random Forest. Lac, LDH, and PLT had the highest importance for the precise prediction of ICU mortality. *ROC* receiver operating characteristic, *AUC* area under the curve, *CI* confidence interval, *ICU* intensive care unit, *APACHE* acute physiology and chronic health evaluation, *SOFA* sequential organ failure assessment, *Lac* lactate, *LDH* lactate dehydrogenase, *PLT* platelet count, *NBPs* non-invasive systolic blood pressure, *UN* urea nitrogen, *cBase (Ecf)* standard base excess, *NBPd* non-invasive diastolic blood pressure, ALP alkaline phosphatase, *cBase (B)* actual base excess, *CRE* creatinine, *HR* heart rate, AST aspartate aminotransferase, *PT-PER* prothrombin time (%), *PR* pulse rate, *WBC* white blood cell, *RR* (impedance) impedance respiratory rate, *PT-SEC* prothrombin time (s).

We first compared predictive values of ICU mortality using three machine learning approaches and logistic regression with acute physiology, and chronic health evaluation (APACHE) II or sequential organ failure assessment (SOFA) score. The prediction algorithm using Random Forest (RF) had the highest predictive value for ICU mortality among the test cohort (Fig. 1a, red line; AUC 0.945 [95% confidence interval: CI 0.922–0.977]) (predictive value among training cohort, Supplementary Table S2). In the analysis of importance using the RF model, lactate, lactate dehydrogenase (LDH), and platelet count were identified as key variables (top three variables) (Fig. 1b). The threshold (mode) of lactate, LDH, and platelet was 2.03 or 10 mmol/L, 301.84 IU/L, and 46.44 10^3^/µL, respectively (Supplementary Fig. S1). The settings of each machine learning model were shown in the additional file (Supplementary Tables S3–S5).

### Cluster analysis of ICU patients

**Figure 2:**
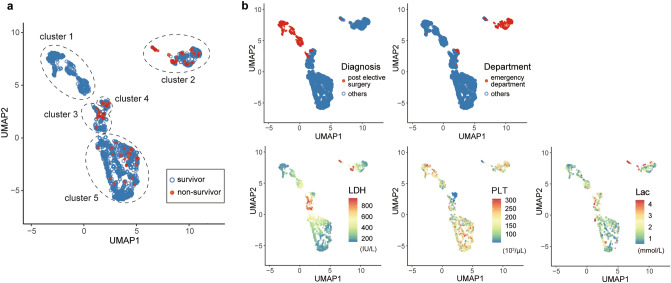
Clustering analysis based on mortality risk in the intensive care unit in the test cohort. (**a**) A clustering with UMAP for ICU patients was performed based on the risk of ICU mortality and the distribution of each variable. The analysis classified the patients into five clusters. (**b**) The top three variables (Lac, LDH, and PLT) contributed to predicting ICU mortality and the other two factors (Diagnosis and Department) characterized each cluster. *UMAP* uniform manifold approximation and projection, *Lac* lactate, *LDH* lactate dehydrogenase, *PLT* platelet count.

To further evaluate the key variables, we performed a clustering analysis in the test cohort. Since the number and shape of clusters depends on the hyperparameter settings, we evaluated several patterns to identify the appropriate setting forming the most visually distinct clusters (Supplementary Fig. S2). The clustering analysis with uniform manifold approximation and projection (UMAP) and the distribution of each variable classified the ICU patients into five clusters based on the risk of ICU mortality (Fig. 2). Clusters 1 and 5 had low mortality rates compared to clusters 2, 3, and 4 (Fig. 2a), and clusters 1 and 2 were characterized by entry route to the ICU (cluster 1, post elective surgeries; cluster 2, from emergency department) (Fig. 2b). In terms of key variables for mortality, cluster 3 had high LDH and cluster 4 had low platelet counts, and high lactate was not localized in specific clusters (Fig. 2b).

### Prediction of the length of ICU stay among survivors

**Figure 3:**
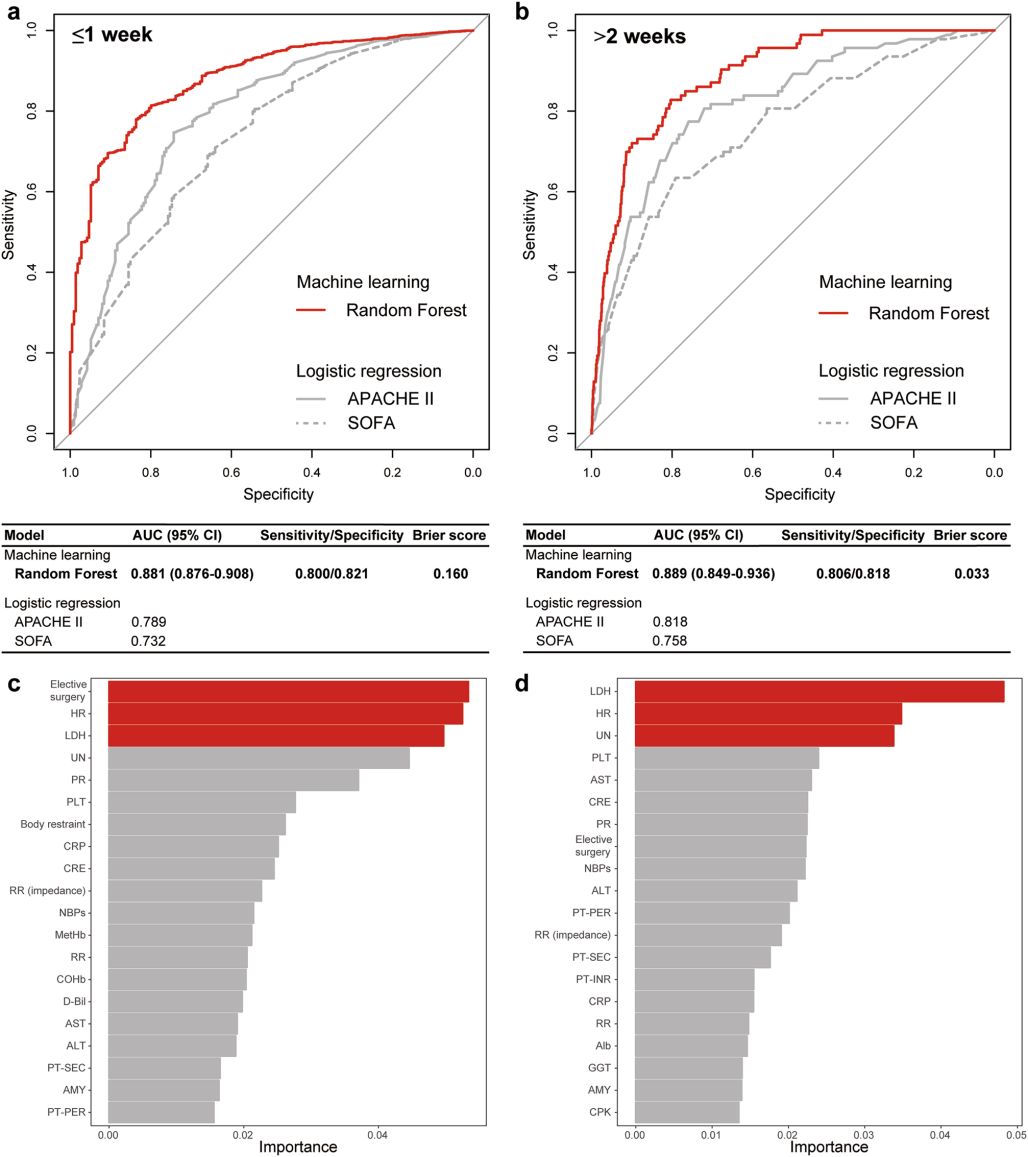
Predictive accuracy and key variables for the length of intensive care unit stay in the test cohort. (**a**,**b**) ROC curves and AUCs for the short (**a**) and long (**b**) length of ICU stays were derived from the machine learning methods using Random Forest and logistic regression. (**c**,**d**) Relative importance of variables for the short (**c**) and long (**d**) length of ICU stays in Random Forest. Elective surgery, HR, and LDH had the highest importance for the precise prediction of short length of ICU stay; LDH, HR, and UN had the highest importance for the precise prediction of long length of ICU stay. *ROC* receiver operating characteristic, *AUC* area under the curve, *CI* confidence interval, *ICU* intensive care unit, *APACHE* acute physiology and chronic health evaluation, *SOFA* sequential organ failure assessment, *HR* heart rate, *LDH* lactate dehydrogenase, *UN* urea nitrogen, *PR* pulse rate, *PLT* platelet count, *CRP* C-reactive protein, *CRE* creatinine, *RR (impedance)* impedance respiratory rate, *NBPs* non-invasive systolic blood pressure, *MetHb* methemoglobin, *RR* respiratory rate (count), *COHb* carboxyhemoglobin, *D-Bil* direct bilirubin, *AST* aspartate aminotransferase, *ALT* alanine aminotransferase, *PT-SEC* prothrombin time (in seconds), *AMY* amylase, *PT-PER* prothrombin time (%), *PT-INR* prothrombin time (international normalized ratio), *Alb* albumin, *GGT* gamma-glutamyltransferase, *CPK* creatine phosphokinase.

We next analyzed the length of ICU stay category prediction. The RF algorithm had better prediction for both short or long stay among survivors compared to the logistic regression analysis models of APACHE II or SOFA score in the test cohort (short/long, AUC 0.881/0.889 [95% CI 0.876–0.908/0.849–0.936]) (Fig. 3a,b) (predictive value among training cohort, Supplementary Table S6). The accuracy of the RF model was 0.961 for short ICU stay and 0.830 for long ICU stay (Supplementary Table S7). Three subgroup predictions using the ordinalForest approach yielded high predictive values in the test cohort (AUCs: short, 0.872 [95% CI 0.853–0.889]; medium, 0.839 [95% CI 0.790–0.880]; long, 0.863 [95% CI 0.835–0.926]) (Supplementary Fig. S3).

The analysis of importance identified the key variables for short ICU stay among survivors to be admission after elective surgery, heart rate (HR), and LDH; for long ICU stay among survivors to be LDH, HR, and urea nitrogen (Fig. 3c,d). For short ICU stay, the threshold (mode) of HR, and LDH was 92.64 or 105.76 bpm, 210.62 or 310.57 IU/L, respectively. For long ICU stay, the threshold (mode) of LDH, HR, and UN was 230.62 IU/L, 75.96 or 94.9 bpm, 16.33 mg/L, respectively (Supplementary Fig. S4). In accordance with the key variables in mortality, LDH was identified as a key variable for both short and long ICU stays among survivors. Prediction of the length of ICU stay was conditional on surviving, therefore we compared the distribution of the important variables shown in Fig. 3 among survivors and non-survivors. Most of these important variables were significantly different between survivors and non-survivors (Supplementary Table S8). Although the above results suggested elective surgery patients had different characteristics, the repeat analysis excluding elective surgery patients showed similar findings (Supplementary Tables S9 and S10, Supplementary Fig. S5).

## Discussion

In the present study, the machine learning algorithms using RF demonstrated a higher predictive value for ICU mortality and length of ICU stay among survivors compared to classical scoring systems. The machine learning prediction indicated that LDH was a contributing variable in precise prediction for both mortality and length of ICU stays among survivors. Cluster analysis with UMAP also identified that LDH contributed to classifying patients into the mortality risk sub-populations.

A unique feature of our study is the examination of key variables to predict ICU mortality and length of ICU stay using machine learning in combination with cluster analysis. LDH was found to be an important predictor of mortality involved in the clustering of ICU patients based on mortality risk and length of ICU stay. LDH is elevated by cellular damage caused by infection, hematologic disease, or liver damage^6^, and has been reported to be associated with mortality in critically ill patients. In septic patients, high LDH (> 225 IU/L) was associated with 28-day mortality; LDH had a favorable predictive value for 28-day mortality among these patients (AUC 0.783)^7^. In acute respiratory distress syndrome patients, high LDH (> 350 IU/L) was associated with increased 60-day mortality^8^, and LDH was the most important variable for predicting mortality (AUC 0.854) among 12 variables (age, sex, pneumonia or not, sepsis or not, white blood cell, albumin, C-reactive protein, LDH, pulmonary surfactant-associated protein D, peptidase inhibitor 3, number of organ failures [none, one, two, and more than two], and PaO_2_/F_I_O_2_)^9^. In acute pancreatitis, patients who died had significantly higher LDH levels (mean 667 IU/L) than survivors (mean 494 IU/L)^10^. In line with these reports, our machine learning investigation found that LDH was a contributing variable in outcome prediction in ICU patients under various conditions.

Our investigation further identified that machine learning had a high predictive value for mortality in ICU patients. Machine learning can analyze a vast amount of data. As ICU patients undergo continuous monitoring of various parameters, they produce a massive amount of data and are thus an optimal target for machine learning^11^. With the development of automatic data collection systems in ICUs^12^, investigations to identify the potential predictive ability for mortality in ICU patients using machine learning have been increasing recently. A previous investigation reported high predictive values for mortality using machine learning in ICU patients (AUC 0.85–0.89)^5,13^. Our results showed similar predictive values to these reports.

In the analysis of the length of ICU stay prediction, we found a remarkable performance of the machine learning algorithm. Prediction of prolonged ICU stay is important for the adequate allocation of medical resources and management of ICU beds^14,15^. Short and long ICU stays are reported to be related to the severity of illness^16^. Thus, precise prediction of short and long ICU stays is essential in the care of critically ill patients. A previous investigation reported that the predictive value for ICU stay longer than 6 days was higher in machine learning algorithms than in SOFA scores (machine learning, AUC 0.76; SOFA score, AUC 0.62)^17^. Patients with ICU stay within 10 days were identified using a machine learning algorithm with an AUC of 0.851^18^. Our investigation identified that the machine learning algorithm may predict both short (within one week) and longer (more than two weeks) ICU stays with high precision (short/long, AUC 0.881/0.889), in line with previous reports. However, in this study, the prediction of the length of ICU stay was conditional on surviving, which should be interpreted with caution. Because many of the important factors for predicting the length of ICU stay differed between survivors and non-survivors, the findings could alter in a population including non-survivors.

The present study has several limitations. First, this was a single-center, retrospective study. In this regard, the prediction accuracy should be validated in a future prospective study. Second, previous investigations were analyzed using data at the earliest timing after ICU admission. Inconsistent timing of sample collection among patients could potentially affect prediction accuracy. Third, we excluded non-survivors to predict the length of ICU stay for competing risks. However, we cannot forecast whether a patient will survive or die at the time of admission, which affects the generalizability of our results to predict the length of ICU stay. Fourth, the prediction was performed based on data collected only at a single time point on admission. Real-time prediction could also be useful to improve the accuracy of prediction of ICU mortality and length of stay of critically ill patients whose condition is subject to abrupt changes. Future investigations using sequential data are warranted.

In conclusions, the machine learning algorithm could predict ICU mortality and short/long length of ICU stay with high accuracy. Moreover, LDH was found to be a key variable predicting both mortality and length of stay, and contributed to the clustering of ICU patients based on mortality risk.

## Methods

### Subjects

This was a retrospective cohort study performed using electronic health record data of consecutive patients admitted to the ICU at Chiba University Hospital, Japan, from November 2010 to March 2019. The surgical/medical ICU has 22 beds, with an annual admission number of patients ranging from 1541 to 1832. Of the 16,169 screened patients, 12,747 were enrolled in the present study after the exclusion of 3,422 with missing data on clinical outcomes.

The study was approved by the Ethical Review Board of the Graduate School of Medicine, Chiba University (approval number: 3380), and performed in accordance with the Declaration of Helsinki. The review board waived the need for written informed consent, in conformity with the Ethical Guidelines for Medical and Health Research Involving Human Subjects in Japan.

### Data collection and definitions

To develop prediction algorithms, the data of 91 input variables (Supplementary Table S11) were collected at the earliest time within 24 h after ICU admission from the ICU data system. These variables included (1) patient baseline characteristics (age, sex, height, weight, blood type, clinical department categories, diagnosis on admission, admission route [from emergency room, general ward, operating room, other hospitals] and APACHE II comorbidities [acquired immunodeficiency syndrome, acute myeloid leukemia/multiple myeloma, heart failure, lymphoma, respiratory failure, cancer metastasis, liver failure/cirrhosis, immunosuppressed status, and dialysis]); (2) blood tests (complete blood count, biochemistry, coagulation, and blood gas analysis); and (3) physiologic measurements (HR, blood pressure, respiratory rate, peripheral oxygen saturation [SpO_2_], and body temperature). Numerical data with an input rate of less than 50% were not used for predictions.

Variable importance is defined as an index calculated by machine learning that indicates how much the model used the variable to make precise predictions. The top three variables with high importance were defined as the key variables in this study. The length of ICU stay was analyzed in survivors and divided into three categories: short (within 1 week), medium (within 1–2 weeks), and long (more than 2 weeks). The short and long length of ICU stay were considered to have high clinical importance because these subcategories were reported to be associated with ICU mortality and severity^16,19^. In addition, identifying patients who are at risk of long ICU stay may contribute to adequate ICU management and avoid ICU bed shortage^16^.

### Imputation for missing values

We performed multiple imputations (10 times) for the missing values of numerical data on single dataset using the *sklearn.impute.Iterative Imputer* in Python (scikit-learn 0.22.1; https://scikit-learn.org). Dummy coding was used to convert categorical variables into binary variables. After missing value imputation, the dataset was randomly split into the training and test cohorts, comprising 80% and 20% of the datasets, respectively, and the variables were compared between the two cohorts.

### Statistical analysis

The primary outcome variable was ICU mortality, and the secondary outcome variable was the length of ICU stay. Outcome prediction was performed using machine learning approach algorithms computed with the three types of classifiers, namely RF, XGBoost, and Neural Network, or logistic regression analysis using either APACHE II score or SOFA score. RF is a standard ensemble machine learning method, and XG Boost is the same decision tree-based method as RF, which has been used frequently in recent years because of its accuracy for complex data. Different from these two classifiers, Neural Network is a non-decision-tree based method. Because it is difficult to evaluate all machine learning methods, these three classifiers, which are representative and have different characteristics, were selected in this study. After machine learning algorithms were derived using the training cohort, the established algorithms were applied to the test cohort. As we found that the RF was superior to the other two machine learning models for the prediction of mortality, we confirmed the variable importance and key variables in the RF model. To evaluate the variable importance in the prediction, we used the feature importances function in Python scikit-learn package.

For robust clustering of ICU patients with higher risk factors for mortality, an RF dissimilarity measure was calculated to evaluate the similarity among patients. The RF dissimilarity measure is a method to evaluate the similarity between samples based on a trained RF model, where the similarity of samples is evaluated by the frequency with which two samples are classified into the same leaf in the decision tree in the RF model^20^. If two samples are classified into the same leaf in all decision trees, the RF dissimilarity between the two samples is 0 (completely same). Conversely, if two samples are never classified into the same leaf, the RF dissimilarity is 1 (completely different). The more often they are classified into the same leaf, the closer the RF dissimilarity is to 0. The RF dissimilarity was then used as an input for UMAP to provide a 2D representation of the patients in the test cohort. UMAP is a type of manifold learning that allows us to place samples in a two-dimensional space while maintaining the distance (dissimilarity) between the samples^21^. Subsequently, clustering of ICU patients was obtained by visually identifying the distribution of each variable on the two scaling coordinates of UMAP. The clustering based on the RF dissimilarity measure that we have done in this study is a visualization of a supervised machine learning model. In supervised learning, the prediction results are probabilities, but the details of the prediction, such as "what samples are likely to be wrong in prediction" or "what samples have similar prediction probabilities but have different characteristics" are not explicitly shown. Visualization and clustering based on the RF dissimilarity allows us to reveal the heterogeneity of the population and hard-to-predict samples.

To predict the length of ICU stay, we evaluated the short (short vs. not short) and long (long vs. not long) categories using machine learning with RF algorithm and logistic regression analysis using the APACHE II or SOFA scores. In the same manner as the analysis on mortality, variable importance and key variables associated with length of ICU stay prediction were confirmed. We also analyzed the predictive values of length of ICU stay using ordinalForest, which could estimate the predictive values for all three categories of ICU stay at the same time. All classifiers were implemented using Python, except for the ordinalForest, which was executed with R.

Data are expressed as median (interquartile range) for continuous values and absolute numbers and percentages for categorical values. The AUC was calculated to evaluate the predictive values. Statistical significance was set at P < 0.05. Analyses were performed using Python packages (sklearn.neural_network.MLPClassifier, sklearn.ensemble.RandomForestClassifier, xgboost, sklearn.linear_model.LogisticRegression) and R package (ordinalForest 2.4.1), to construct machine learning models.

### Ethics approval and consent to participate

This study was approved by the research ethics committee of the Chiba University Graduate School of Medicine (approval number: 3380), who issued a waiver for written consent for the study because data collection was retrospective.

## Supplementary Information

Supplementary Information.

## Data Availability

The datasets used and analyzed during the current study are available from the corresponding author upon reasonable request.

## Abbreviations

APACHE: Acute physiology and chronic health evaluation
AUC: Area under the curve
CI: Confidence interval
HR: Heart rate
ICU: Intensive care unit
LDH: Lactate dehydrogenase
RF: Random forest
SOFA: Sequential organ failure assessment
SpO_2_: Peripheral oxygen saturation
UMAP: Uniform manifold approximation and projection

## Code availability

The Python and R codes used in this article are available at https://github.com/eiryo-kawakami/ICU_AI_code.
