# Supplementary material for: Prediction algorithm for ICU mortality and length of stay using machine learning
Source: Sci Rep. 2022 Jul 28;12:12912. doi: 10.1038/s41598-022-17091-5 (PMC9334583; doi:10.1038/s41598-022-17091-5)
Supplement: Supplementary file 1 — Supplementary Information. [file 41598_2022_17091_MOESM1_ESM.docx]

**Supplementary Information**

Iwase S, Nakada TA, Shimada T, Oami T, Shimazui T, Takahashi N, Yamabe J, Yamao Y, Kawakami E

Prediction algorithm for ICU mortality and length of stay using machine learning

**Supplementary Table S1.** Baseline characteristics of patients in the training and test cohorts

**Supplementary Table S2.** Predictive accuracy for intensive care unit mortality in the training set

**Supplementary Table S3.** Hyperparameters for Random Forest

**Supplementary Table S4.** Hyperparameters for XGBoost

**Supplementary Table S5.** Hyperparameters for Neural Network

**Supplementary Table S6.** Predictive accuracy for short or long intensive care unit stay in the training set

**Supplementary Table S7.** Accuracy score of the prediction by Random Forest

**Supplementary Table S8.** Distribution of important variables for predicting length of intensive care unit stay among survivors and non-survivors

**Supplementary Table S9.** Predictive accuracy for intensive care unit mortality among patients without elective surgery in the test set

**Supplementary Table S10.** Predictive accuracy for short or long intensive care unit stay among patients without elective surgery in the test set

**Supplementary Table S11.** Input variables

**Supplementary Figure S1.** Threshold distribution of key variables for mortality prediction in Random Forest

**Supplementary Figure S2.** Clustering changes in several hyperparameter settings

**Supplementary Figure S3.** Predictive accuracy of ordinalForest for the length of intensive care unit stay

**Supplementary Figure S4.** Threshold distribution of key variables for the length of intensive care unit stay prediction

**Supplementary Figure S5.** Key variables for mortality and the length of intensive care unit stay among patients without elective surgery in the test set

**Supplementary Table S1. Baseline characteristics of patients in the training and test cohorts**

| **Variables** | **Training cohort** | **Test cohort** | **P-value** |
| --- | --- | --- | --- |
|  | (n=10,197) | (n=2,550) |  |
| Demographic data |  |  |  |
| Height, cm | 161.4 (154.0-168.0) | 161.1 (153.1-167.8) | 0.84 |
| Weight, kg | 58.0 (49.8-67.0) | 57.7 (49.5-66.2) | 0.40 |
| Blood type |  |  |  |
| ABO |  |  |  |
| Type A | 3,379 (33.1) | 845 (33.1) | 1 |
| Type B | 2,048 (20.1) | 555 (21.8) | 0.06 |
| Type O | 2,854 (28.0) | 690 (27.1) | 0.35 |
| Type AB | 905 (8.9) | 195 (7.7) | 0.05 |
| Rh |  |  |  |
| Type + | 9,148 (89.7) | 2,285 (89.6) | 0.88 |
| Department |  |  |  |
| Emergency medicine | 1,671 (16.4) | 415 (16.3) | 0.89 |
| Cardiovascular surgery | 1,728 (17.0) | 405 (15.9) | 0.20 |
| Cardiology | 1,788 (17.5) | 472 (18.5) | 0.25 |
| Surgery | 1,800 (17.7) | 434 (17.0) | 0.45 |
| Neurosurgery | 1,305 (12.8) | 323 (12.7) | 0.86 |
| Others | 1,905 (18.7) | 501 (19.6) | 0.27 |
| Medical emergency team call | 183 (1.8) | 44 (1.7) | 0.81 |
| Transport method |  |  |  |
| Ambulance | 737 (7.2) | 189 (7.4) | 0.75 |
| Helicopter | 77 (0.8) | 21 (0.8) | 0.72 |
| Admission time |  |  |  |
| 09:00 a.m. - 16:59 p.m. | 4,878 (47.3) | 1,204 (47.2) | 0.57 |
| 17:00 p.m. - 00:59 a.m. | 4,383 (43.0) | 1,123 (44.0) | 0.34 |
| 01:00 a.m. - 08:59 a.m. | 936 (9.2) | 223 (8.7) | 0.50 |
| Body restraint | 1,159 (11.4) | 295 (11.6) | 0.77 |
| Blood test |  |  |  |
| Complete blood count |  |  |  |
| WBC, 10^3^/μL | 10.0 (7.4-13.3) | 10.0 (7.5-13.5) | 0.53 |
| RBC, 10^6^/μL | 3.51 (3.05-4.04) | 3.53 (3.07-4.05) | 0.23 |
| HGB, g/dL | 10.7 (9.4-12.3) | 10.8 (9.4-12.4) | 0.19 |
| HCT, % | 31.7 (27.6-36.4) | 32.1 (27.8-36.7) | 0.08 |
| MCV, fL | 90.6 (86.8-94.6) | 90.9 (86.9-94.9) | 0.17 |
| MCH, pg | 30.9 (29.7-32.0) | 30.9 (29.7-32.0) | 0.99 |
| MCHC, % | 33.9 (33.0-34.8) | 33.9 (32.9-34.7) | 0.10 |
| Platelet, 10^3^/μL | 162 (108-216) | 163 (110-213) | 0.48 |
| Segmented neutrophil, % | 83.8 (74.8-89.2) | 83.9 (75.1-89.4) | 0.59 |
| Eosinophil, % | 0.1 (0.0-0.6) | 0.1 (0.0-0.5) | 0.81 |
| Basophil, % | 0.1 (0.0-0.2) | 0.1 (0.0-0.2) | 0.72 |
| Monocyte, % | 4.8 (3.2-6.4) | 4.7 (3.3-6.3) | 0.82 |
| Lymphocyte, % | 7.8 (4.5-13.3) | 7.8 (4.5-13.5) | 0.72 |
| Blood chemistry |  |  |  |
| TP, g/dL | 5.4 (4.7-6.1) | 5.4 (4.8-6.1) | 0.67 |
| Albumin, g/dL | 3.1 (2.6-3.5) | 3.1 (2.6-3.5) | 0.49 |
| AST, IU/L | 36 (22-79) | 38 (22-84) | 0.20 |
| ALT, IU/L | 21 (13-47) | 22 (14-49) | 0.31 |
| LDH, IU/L | 259 (192-388) | 259 (190-401) | 0.08 |
| ALP, IU/L | 180 (134-254) | 185 (136-263) | 0.02 |
| GGT, IU/L | 27 (16-55) | 28.5 (17-61) | 0.03 |
| T-Bil, mg/dL | 0.9 (0.6-1.4) | 0.9 (0.6-1.4) | 0.73 |
| D-Bil, mg/dL | 0.1 (0.1-0.3) | 0.1 (0.1-0.3) | 0.55 |
| Amylase, IU/L | 87 (52-184) | 91 (56-187) | 0.07 |
| CPK, IU/L | 253 (83-748) | 264 (81-780) | 0.71 |
| UN, mg/dL | 16 (11-23) | 16 (12-23) | 0.93 |
| Creatinine, mg/dL | 0.80 (0.61-1.17) | 0.79 (0.61-1.16) | 0.40 |
| UA, mg/dL | 4.8 (3.4-6.3) | 4.7 (3.5-6.2) | 0.85 |
| Na, mEq/L | 139 (137-141) | 139 (137-141) | 0.53 |
| K, mEq/L | 4.0 (3.8-4.4) | 4.1 (3.8-4.4) | 0.02 |
| Cl, mEq/L | 107 (104-109) | 106 (104-109) | 0.98 |
| Ca, mg/dL | 8.0 (7.5-8.6) | 8.0 (7.5-8.6) | 0.69 |
| CRP, mg/dL | 3.2 (0.8-7.0) | 3.3 (0.9-7.3) | 0.16 |
| Coagulation |  |  |  |
| PT-SEC, sec | 11.7 (10.9-13.0) | 11.6 (10.9-12.9) | 0.14 |
| PT-PER, % | 84.0 (64.0-98.0) | 84.0 (67.0-98.0) | 0.22 |
| PT-INR | 1.08 (1.01-1.20) | 1.08 (1.01-1.19) | 0.18 |
| Blood gas analysis |  |  |  |
| pH | 7.42 (7.39-7.46) | 7.42 (7.38-7.46) | 0.93 |
| PCO_2_, mmHg | 40 (36-44) | 40 (36-44) | 0.60 |
| PO_2_, mmHg | 116 (88-153) | 116 (88-153) | 0.60 |
| O_2_Hb, % | 96.4 (95.6-97.1) | 96.4 (95.6-97.2) | 0.63 |
| SO_2_, % | 97.7 (96.7-99.0) | 97.6 (96.6-98.9) | 0.66 |
| SO_2_ (c), % | 98.8 (97.0-99.2) | 98.7 (97.0-99.2) | 0.51 |
| THb, g/dL | 10.4 (9.3-11.8) | 10.5 (9.2-11.9) | 0.83 |
| HHb, g/dL | 2.3 (1.0-3.3) | 2.3 (1.1-3.4) | 0.64 |
| THb (c), g/dL | 9.9 (8.5-11.5) | 9.9 (8.5-11.5) | 0.76 |
| Hct, % | 30.0 (26.0-35.0) | 30.0 (26.0-36.0) | 0.82 |
| MetHb, % | 0.6 (0.3-1.0) | 0.6 (0.3-1.0) | 0.37 |
| COHb, % | 0.3 (0.0-1.4) | 0.3 (0.0-1.3) | 0.92 |
| HCO_3_^-^, mmol/L | 25.9 (23.7-27.9) | 25.9 (23.6-28.2) | 0.70 |
| cBase (B), mmol/L | 1.3 (-0.8-3.4) | 1.2 (-0.9-3.5) | 0.92 |
| cBase (Ecf), mmol/L | 1.4 (-1.1-3.8) | 1.4 (-1.1-3.9) | 0.83 |
| AG, mmol/L | 9.0 (7.0-11.0) | 9.0 (7.0-11.0) | 0.69 |
| Na^+^, mmol/L | 137 (135-140) | 137 (135-140) | 0.52 |
| K^+^, mmol/L | 3.9 (3.6-4.2) | 3.9 (3.6-4.3) | 0.06 |
| Cl^-^, mmol/L | 107 (105-110) | 108 (104-110) | 0.37 |
| Ca^2+^, mmol/L | 1.13 (1.09-1.17) | 1.13 (1.08-1.17) | 0.25 |
| Lactate, mmol/L | 1.3 (0.9-1.9) | 1.2 (0.9-1.9) | 0.90 |
| Glucose, mg/dL | 137 (114-166) | 138 (114-167) | 0.84 |
| Physiologic measurements |  |  |  |
| Heart rate, bpm | 86 (74-101) | 86 (74-101) | 0.78 |
| Pulse rate, bpm | 85 (73-99) | 85 (73-99) | 0.88 |
| Blood pressure, mmHg |  |  |  |
| NBP systolic | 123 (105-141) | 122 (105-141) | 0.70 |
| NBP diastolic | 70 (59-81) | 69 (59-81) | 0.57 |
| ABP systolic | 127 (104-150) | 126 (104-151) | 0.12 |
| ABP mean | 84 (70-98) | 85 (70-100) | 0.04 |
| ABP diastolic | 62 (52-72) | 63 (53-73) | 0.02 |
| Respiratory rate, bpm |  |  |  |
| Impedance | 18 (15-22) | 18 (15-22) | 0.56 |
| Count | 18 (15-22) | 18 (15-22) | 0.39 |
| Oxygen saturation, % | 99 (97-100) | 99 (97-100) | 0.20 |
| Body temperature, ℃ | 36.7 (36.3-37.3) | 36.7 (36.3-37.2) | 0.71 |

WBC, white blood cell; RBC, red blood cell; HGB, hemoglobin; HCT, hematocrit; MCV, mean corpuscular volume; MCH, mean corpuscular hemoglobin; MCHC, mean corpuscular hemoglobin concentration; TP, total protein; AST, aspartate aminotransferase; ALT, alanine aminotransferase; LDH, lactate dehydrogenase; ALP, alkaline phosphatase; GGT, gamma-glutamyltransferase; T-Bil; total bilirubin; D-Bil, direct bilirubin; CPK, creatine phosphokinase; UN, urea nitrogen; UA, uric acid; CRP, C-reactive protein; PT-SEC, prothrombin time (in seconds); PT-PER, prothrombin time (%); PT-INR, prothrombin time (international normalized ratio); O_2_Hb, oxyhemoglobin; SO_2_, oxygen saturation; SO_2_ (c), calculated oxygen saturation; THb, total hemoglobin; HHb, deoxyhemoglobin; THb (c), calculated total hemoglobin; Hct, hematocrit; MetHb, methemoglobin; COHb, carboxyhemoglobin; cBase (B), actual base excess; cBase (Ecf), standard base excess; AG, anion gap; NBP, non-invasive blood pressure; ABP, arterial blood pressure

Data are expressed as median (interquartile range) for continuous variables and as exact numbers (%) for categorical variables.

P-values were calculated using Pearson’s chi-square test and Mann-Whitney U test.

**Supplementary Table S2. Predictive accuracy for intensive care unit mortality in the training set**

| **Model** | **AUC** |
| --- | --- |
| Random Forest | 0.989 |
| XGBoost | 0.993 |
| Neural Network | 0.955 |

All predictions for mortality using machine learning were highly accurate.

AUC, area under the curve

**Supplementary Table S3. Hyperparameters for RandomForest**

|  | **mortality** | **short stay** | **long stay** |
| --- | --- | --- | --- |
| bootstrap | True | True | True |
| ccp_alpha | 0.0 | 0.0 | 0.0 |
| class_weight | None | ‘balanced’ | ‘balanced’ |
| criterion | ‘entropy’ | ‘entropy’ | ‘gini’ |
| max_depth | None | 5 | 15 |
| max_features | 62 | 8 | 14 |
| max_leaf_nodes | None | None | None |
| max_samples | None | None | None |
| min_impurity_decrease | 0.0 | 0.0 | 0.0 |
| min_impurity_split | None | None | None |
| min_samples_leaf | 1 | 1 | 1 |
| min_samples_split | 2 | 2 | 2 |
| min_weight_fraction_leaf | 0.0 | 0.0 | 0.0 |
| n_estimators | 1,000 | 1,000 | 1,000 |
| n_jobs | 6 | 6 | 6 |
| oob_score | False | False | False |
| random_state* | i | i | i |
| verbose | 0 | 0 | 0 |
| warm_start | False | False | False |

* Ten independent training times were performed using different ‘random_state’, and the average of the ten times training was used for accuracy and variable importance.

**Supplementary Table S4. Hyperparameters for XGBoost**

|  | **mortality** | **short stay** | **long stay** |
| --- | --- | --- | --- |
| base_score | 0.5 | 0.5 | 0.5 |
| booster | ‘gbtree’ | ‘gbtree’ | ‘gbtree’ |
| colsample_bylevel | 1 | 1 | 1 |
| colsample_bynode | 1 | 1 | 1 |
| colsample_bytree | 1 | 1 | 1 |
| gamma | 0 | 0 | 0 |
| learning_rate | 0.1 | 0.1 | 0.1 |
| max_delta_step | 0 | 0 | 0 |
| max_depth | 10 | 6 | 10 |
| min_child_weight | 1 | 1 | 1 |
| missing | None | None | None |
| n_estimators | 1,000 | 1,000 | 1,000 |
| n_jobs | 6 | 6 | 6 |
| nthread | None | None | None |
| objective | ‘binary:logistic’ | ‘binary:logistic’ | ‘binary:logistic’ |
| random_state* | i | i | i |
| reg_alpha | 1 | 1 | 0 |
| reg_lambda | 1 | 1 | 1 |
| scale_pos_weight | 1 | 1 | 1 |
| seed | None | None | None |
| silent | None | None | None |
| subsample | 0.9 | 0.9 | 0.9 |
| verbosity | 0 | 0 | 0 |

* Ten independent training times were performed using different ‘random_state’, and the average of the ten times training was used for accuracy and variable importance.

**Supplementary Table S5. Hyperparameters for NeuralNetwork**

|  | **mortality** | **short stay** | **long stay** |
| --- | --- | --- | --- |
| activation | ‘tanh’ | ‘tanh’ | ‘tanh’ |
| beta_1 | 0.9 | 0.9 | 0.9 |
| beta_2 | 0.99 | 0.99 | 0.99 |
| epsilon | 1e-09 | 1e-09 | 1e-09 |
| max_iter | 1,000 | 1,000 | 1,000 |
| momentum | 0.9 | 0.9 | 0.9 |
| random_state* | i | i | i |
| warm_start | True | True | True |
| verbose | 2 | 2 | 2 |

* Ten independent training times were performed using different ‘random_state’, and the average of the ten times training was used for accuracy and variable importance.

**Supplementary Table S6. Predictive accuracy for short or long intensive care unit stay in the training set**

| **Model** | **AUC (short/long)** |
| --- | --- |
| Random Forest | 0.848/0.987 |
| XGBoost | 0.989/0.995 |
| Neural Network | 0.907/0.959 |

Short, within one week; long, more than two weeks

**Supplementary Table S7. Accuracy score of the prediction by RandomForest**

|  | **mortality** | **short stay** | **long stay** |
| --- | --- | --- | --- |
| accuracy score | 0.967 | 0.961 | 0.830 |

**Supplementary Table S8. Distribution of important variables for predicting length of intensive care unit stay among survivors and non-survivors**

| **Variables** | **Survivor** | **Non-survivor** | **P-value** |
| --- | --- | --- | --- |
|  | (n=12,133) | (n=614) |  |
| Demographic data |  |  |  |
| Elective surgery | 3,385 (27.9) | 19 (3.1) | < 0.001 |
| Body restraint | 1,385 (11.4) | 69 (11.2) | 0.89 |
| Blood test |  |  |  |
| LDH, IU/L | 253 (189-375) | 521 (319-1201) | < 0.001 |
| UN, mg/dL | 16 (11-23) | 25 (16-41) | < 0.001 |
| Platelet, 10^3^/µL | 164 (112-217) | 101 (45-176) | < 0.001 |
| CRP, mg/dL | 3.2 (0.8-6.9) | 4.1 (0.8-10.5) | < 0.001 |
| Creatinine, mg/dL | 0.79 (0.60-1.12) | 1.34 (0.83-2.10) | < 0.001 |
| AST, IU/L | 36 (22-76) | 97 (38-482) | < 0.001 |
| ALT, IU/L | 21 (13-44) | 54 (24-204) | < 0.001 |
| GGT, IU/L | 21 (13-44) | 54 (24-204) | < 0.001 |
| D-Bil, mg/dL | 0.1 (0.1-0.2) | 0.2 (0.1-0.9) | < 0.001 |
| PT-SEC, sec | 11.6 (10.9-12.9) | 13.6 (11.9-17.4) | < 0.001 |
| PT-PER, % | 85 (67-99) | 59 (36-82) | < 0.001 |
| PT-INR | 1.08 (1.01-1.18) | 1.25 (1.10-1.57) | < 0.001 |
| Albumin, g/dL | 3.1 (2.7-3.5) | 2.7 (2.2-3.4) | < 0.001 |
| CPK, IU/L | 256 (83-747) | 245 (72-1109) | < 0.001 |
| Amylase, IU/L | 87 (53-182) | 98 (54-219) | 0.54 |
| MetHb, % | 0.6 (0.3-1.0) | 0.6 (0.2-1.0) | 0.01 |
| COHb, % | 0.3 (0.0-1.4) | 0.5 (0.0-1.4) | 0.01 |
| Physiologic measurements |  |  |  |
| Heart rate, bpm | 86 (74-100) | 100 (79-121) | < 0.001 |
| Pulse rate, bpm | 85 (73-98) | 96 (76-118) | < 0.001 |
| RR (impedance), bpm | 18 (15-22) | 22 (16-28) | < 0.001 |
| NBPs, mmHg | 124 (106-142) | 105 (83-130) | < 0.001 |
| RR (count), bpm | 18 (15-22) | 21 (16-27) | < 0.001 |

LDH, lactate dehydrogenase; UN, urea nitrogen; CRP, C-reactive protein; AST, aspartate aminotransferase; ALT, alanine aminotransferase; GGT, gamma-glutamyltransferase; D-Bil, direct bilirubin; PT-SEC, prothrombin time (in seconds); PT-PER, prothrombin time (%); PT-INR, prothrombin time (international normalized ratio); CPK, creatine phosphokinase; MetHb, methemoglobin; COHb, carboxyhemoglobin; NBPs, non-invasive systolic blood pressure; RR, respiratory rate

Data are expressed as median (interquartile range) for continuous variables and as exact numbers (%) for categorical variables.

P-values were calculated using Pearson’s chi-square test and Mann-Whitney U test.

**Supplementary Table S9. Predictive accuracy for intensive care unit mortality among patients without elective surgery in the test set**

| **Model** | **AUC** |
| --- | --- |
| Random Forest | 0.929 |
| XGBoost | 0.928 |
| Neural Network | 0.821 |

**Supplementary Table S10. Predictive accuracy for short or long intensive care unit stay among patients without elective surgery in the test set**

| **Model** | **AUC (short/long)** |
| --- | --- |
| Random Forest | 0.859/0.842 |
| XGBoost | 0.855/0.871 |
| Neural Network | 0.735/0.756 |

Short, within one week; long, more than two weeks

**Supplementary Table 11. Input variables**

| **Baseline characteristics** |
| --- |
| Age (99.9), gender (100), height (70.2), weight (73.1), blood type (ABO and Rh) (90.2), department (99.9), diagnosis on admission (62.3), admission route (47.2), medical emergency team call* (100), transport method (8.7), admission time (100), APACHE II comorbidities (acquired immunodeficiency syndrome, acute myeloid leukemia/multiple myeloma, heart failure, lymphoma, respiratory failure, cancer metastasis, liver failure, cirrhosis, immunosuppressed status, and dialysis)* (100), and body restraint* (100) |
| **Blood tests** |
| Complete blood count (WBC (98.0), RBC (98.1), HGB (98.1), HCT (98.1), MCV (98.1), MCH (98.1), MCHC (98.1), platelet (98.0), Seg (69.9), Eo (69.9), Ba (69.9), monocyte (69.9), and lymphocyte (69.6)), biochemistry (TP (95.4), Alb (96.0), AST (97.1), ALT (97.8), LDH (96.6), ALP (94.4), GGT (90.1), T-Bil (94.7), D-Bil (94.4), AMY (52.2), CPK (80.4), UN (97.8), CRE (97.7), UA (72.7), Na (97.3), K (96.5), Cl (97.3), Ca (78.8), and CRP (96.5)), coagulation (PT-SEC (78.0), PT-PER (78.0), and PT-INR (78.0)), and blood gas analysis (pH (80.9), PCO_2_ (80.9), PO_2_ (80.8), O_2_Hb (64.2), SO_2_ (64.2), SO_2_ (c) (80.7), THb (64.3), HHb (64.2), THb (c) (80.6), Hct (80.8), MetHb (64.2), COHb (64.2), HCO_3_^-^ (80.7), cBase (B) (80.7), cBase (Ecf) (80.7), AG (64.8), Na^+^ (80.8), K^+^ (80.9), Cl^-^ (64.9), Ca^2+^ (80.8), lactate (80.9), and glucose (80.8)) |
| **Physiologic measurements** |
| Heart rate (99.8), pulse rate (99.7), blood pressure (noninvasive systolic (99.3)/diastolic (99.3) and invasive systolic (72.7)/mean (72.7)/diastolic (72.7)), respiratory rate (impedance (80.4) and count (98.2)), oxygen saturation (99.7), and body temperature (97.2) |

(Input rate, %)

APACHE, acute physiology and chronic health evaluation; WBC, white blood cell; RBC, red blood cell; HGB, hemoglobin; HCT, hematocrit; MCV, mean corpuscular volume; MCH, mean corpuscular hemoglobin; MCHC, mean corpuscular hemoglobin concentration; Seg, segmented neutrophil; Eo, eosinophil; Ba, basophil; TP, total protein; Alb, albumin; AST, aspartate aminotransferase; ALT, alanine aminotransferase; LDH, lactate dehydrogenase; ALP, alkaline phosphatase; GGT, gamma-glutamyltransferase; T-Bil; total bilirubin; D-Bil, direct bilirubin; AMY, amylase; CPK, creatine phosphokinase; UN, urea nitrogen; CRE, creatinine; UA, uric acid; CRP, C-reactive protein; PT-SEC, prothrombin time (in seconds); PT-PER, prothrombin time (%); PT-INR, prothrombin time (international normalized ratio); O_2_Hb, oxyhemoglobin; SO_2_, oxygen saturation; SO_2_ (c), calculated oxygen saturation; THb, total hemoglobin; HHb, deoxyhemoglobin; THb (c), calculated total hemoglobin; Hct, hematocrit; MetHb, methemoglobin; COHb, carboxyhemoglobin; cBase (B), actual base excess; cBase (Ecf), standard base excess; AG, anion gap

***** These variables were treated as not having these characteristics if they were missing.

**Supplementary Figure S1.** **Threshold distribution of key variables for mortality prediction in Random Forest**

The threshold (mode) of lactate, LDH, and platelet was 2.03 or 10 mmol/L, 301.84 IU/L, and 46.44 10^3^/µL, respectively.


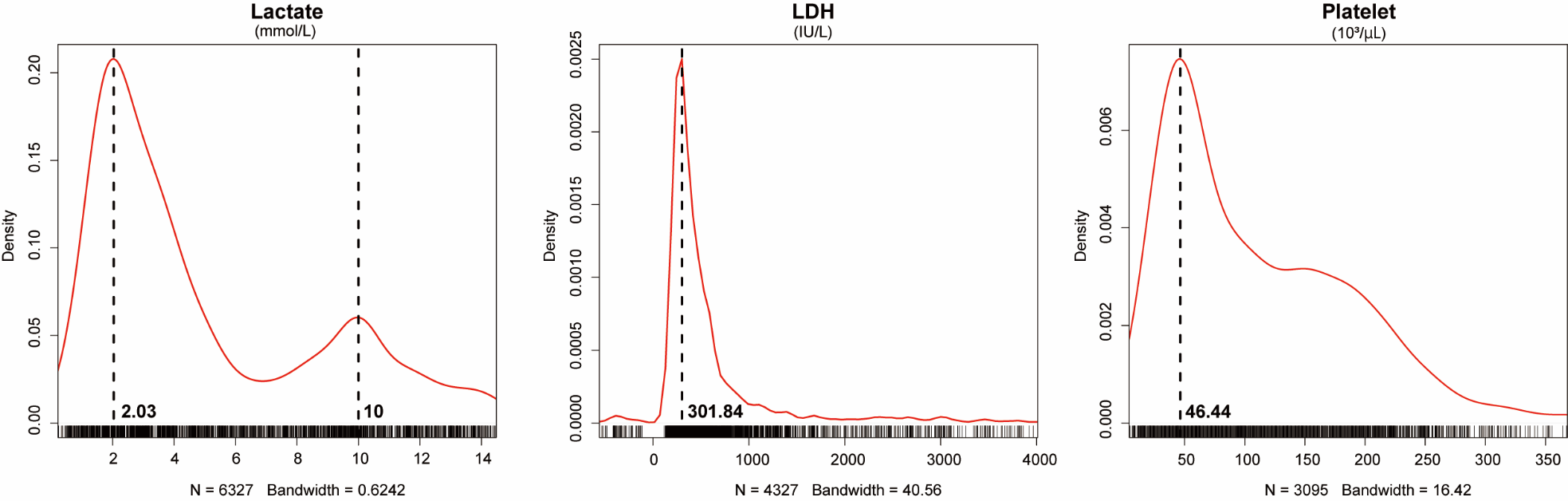


LDH, lactate dehydrogenase

**Supplementary Figure S2. Clustering changes in several hyperparameter settings**

(a) depth = None

(b) depth = 3

(c) depth = 5

(d) depth = 10


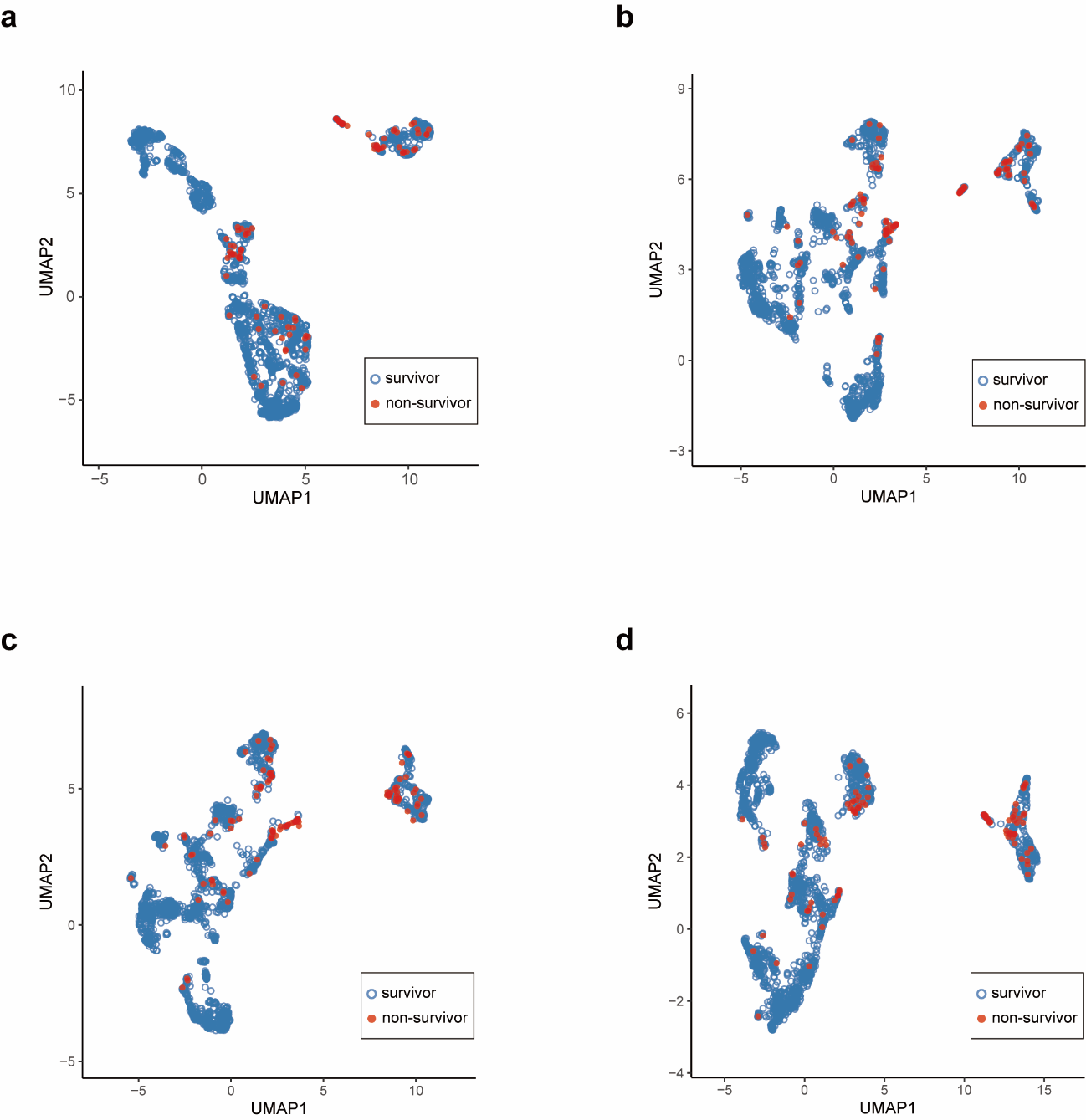


**Supplementary Figure S3. Predictive accuracy of ordinalForest for the length of intensive care unit stay**


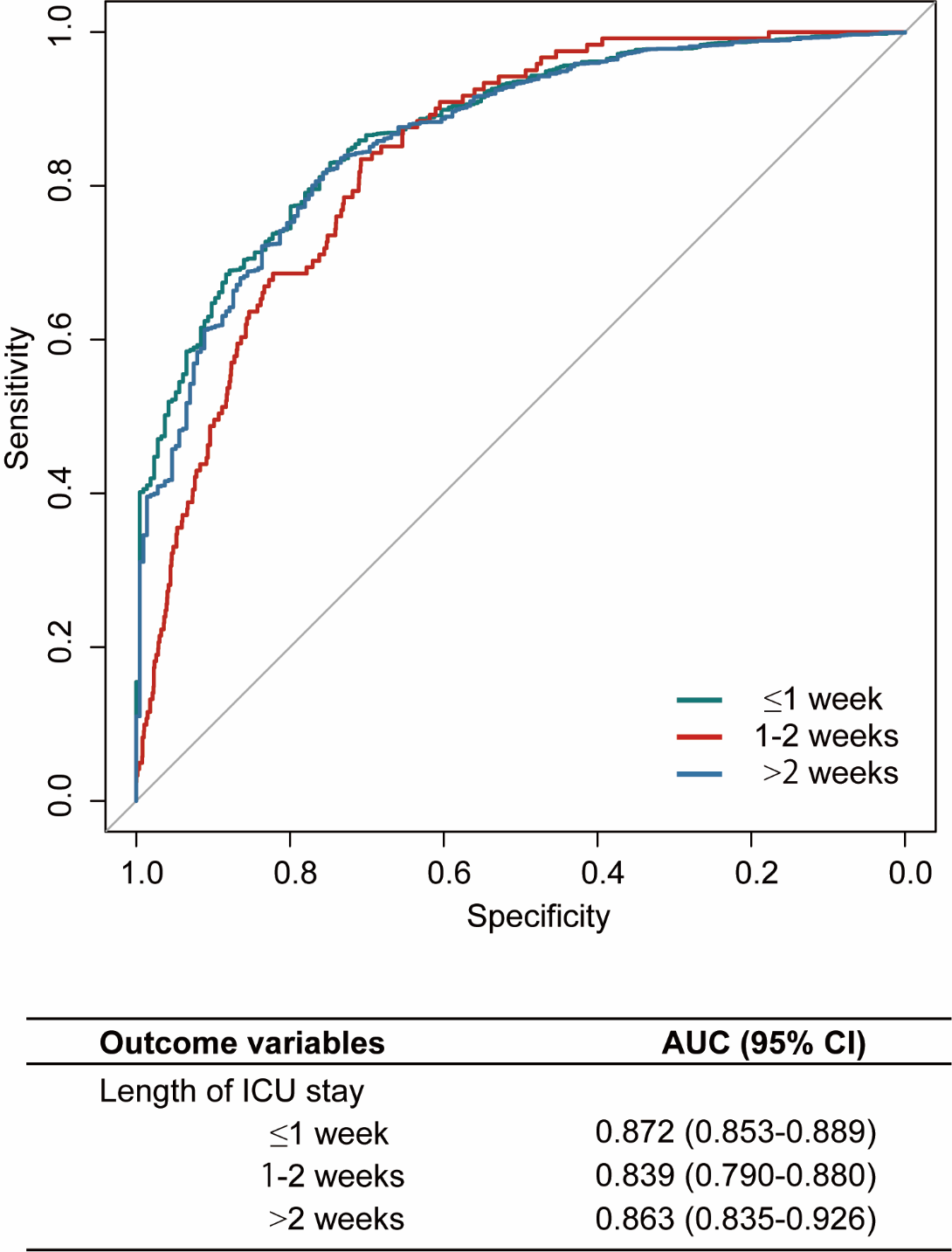


ROC curves and AUCs for the length of ICU stay were derived from ordinalForest.

ROC, receiver operating characteristic; AUC, area under the curve; ICU, intensive care unit; CI, confidence interval

**Supplementary Figure S4. Threshold distribution of key variables for the length of intensive care unit stay prediction**

(a) Threshold distribution of key variable for short ICU stay prediction. The threshold (mode) of HR, and LDH was 92.64 or 105.76 bpm, 210.62 or 310.57 IU/L, respectively.

(b) Threshold distribution of key variable for long ICU stay prediction. The threshold (mode) of LDH, HR, and UN was 230.62 IU/L, 75.96 or 94.9 bpm, 16.33 mg/L, respectively.


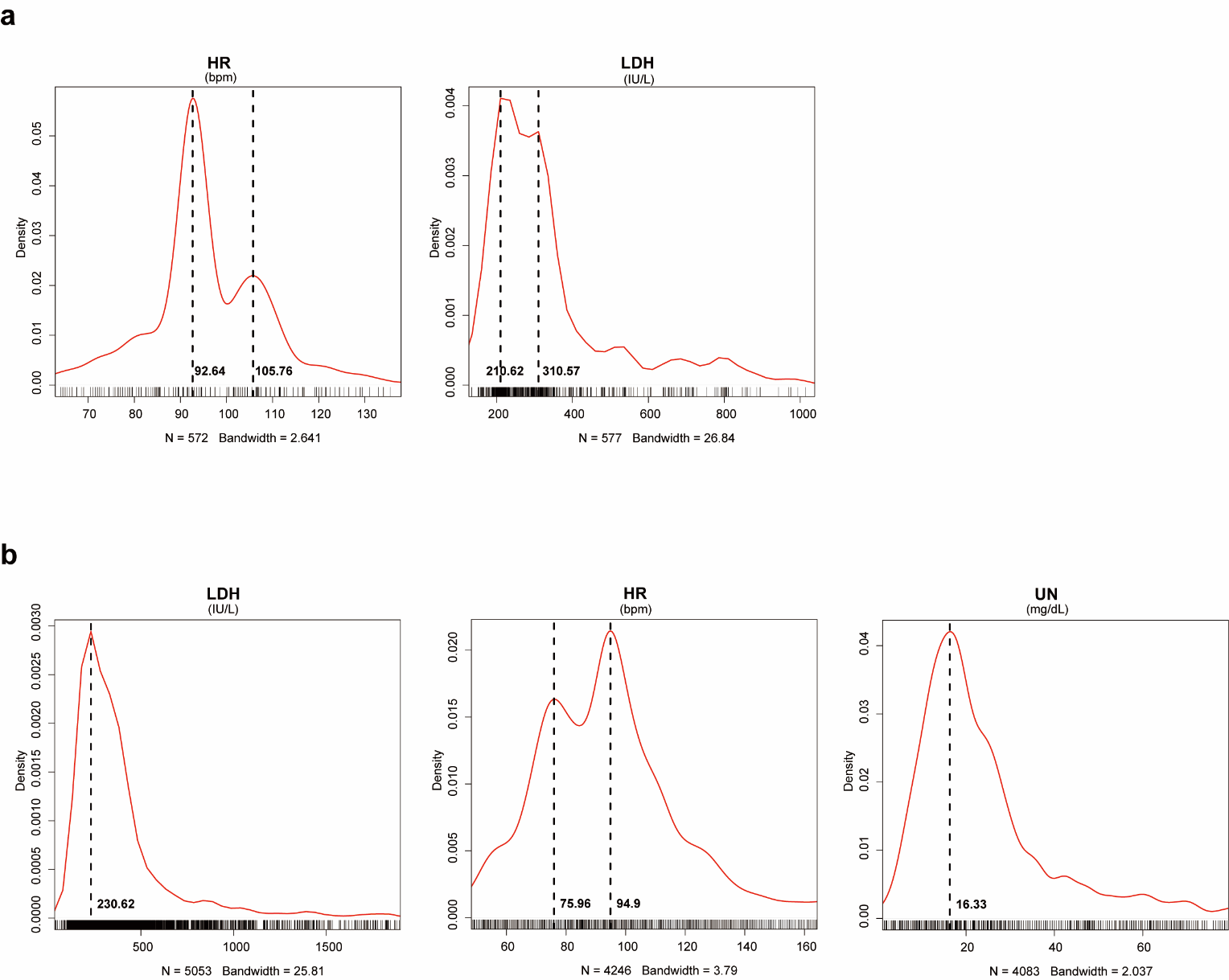


HR, heart rate; LDH, lactate dehydrogenase; UN, urea nitrogen

**Supplementary Figure S5.** **Key variables for mortality and the length of intensive care unit stay among patients without elective surgery in the test set**

(a) Relative importance of variables for ICU mortality in Random Forest. Lac, LDH, and PLT had the highest importance for the precise prediction of ICU mortality.

(b, c) Relative importance of variables for the short (b) and long (c) length of ICU stays in Random Forest. LDH, UN, and HR had the highest importance for the precise prediction of both short and long length of ICU stay.

These results were similar to predictions included elective surgery patients.


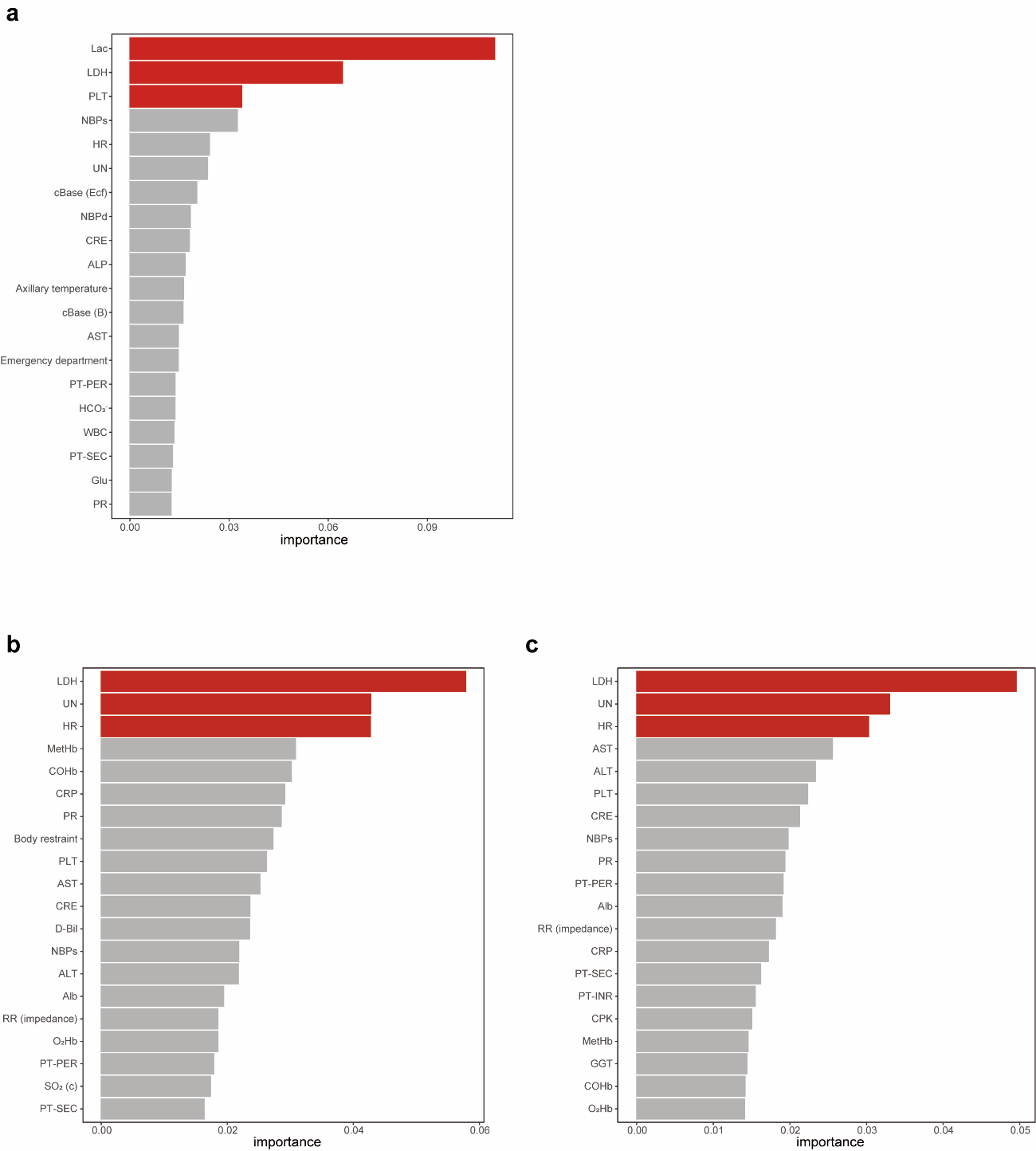


Lac, lactate; LDH, lactate dehydrogenase; PLT, platelet count; NBPs, non-invasive systolic blood pressure; HR, heart rate; UN, urea nitrogen; cBase (Ecf), standard base excess; NBPd, non-invasive diastolic blood pressure; CRE, creatinine; ALP, alkaline phosphatase; cBase (B), actual base excess; AST, aspartate aminotransferase; PT-PER, prothrombin time (%); WBC, white blood cell; Glu, Glucose; PR, pulse rate; MetHb, methemoglobin; COHb, carboxyhemoglobin; CRP, C-reactive protein; D-Bil, direct bilirubin; ALT, alanine aminotransferase; Alb, albumin; RR (impedance), impedance respiratory rate; O_2_Hb, oxyhemoglobin; SO_2_ (c), calculated oxygen saturation; PT-SEC, prothrombin time (in seconds); PT-INR, prothrombin time (international normalized ratio); CPK, creatine phosphokinase; GGT, gamma-glutamyltransferase
